# Medical Education at Constantinople

**Published:** 1848-01

**Authors:** 


*Medical Education at Constantinople.*—The medical school of Galata, Constantinople, is in a thriving condition. The courses of lectures are at present attended by 454 pupils, of whom 409 are matriculated students. The periodical examinations are rigorously conducted, and every student who, after two years' study, does not reach the upper class, is thereafter excluded. Sixty pupils have been expelled during the present year, either for ignorance or misconduct. There are ten classes of students, and more than one half (238) are placed in the first class.

It is a remarkable fact, that Chairs of Medical Jurisprudence and Police are already established in this infant institution; and that Turkey is in this respect in advance of England, is proved by the fact, that, in the event of murder, there is a government medical officer specially appointed to conduct the inspection and report on the cause of death. The Royal College of Surgeons of England, it is well known, does not consider a knowledge of these subjects to be of any importance to its members, or of any utility to society: hence the study of them does not enter into its curriculum! The Government, too, leaves the selection of medical men for the purpose of making inspections and reporting on the cause of death, in cases of suspected murder, to parochial beadles and the local constabulary! The power of ordering post-mortem examinations is also left in the hands of a functionary who is in most cases a non-professional man!" —*Medical Gazette.*

